# Characterization of Ancient Cereals Cultivated by Intensive and Organic Procedures for Element Content

**DOI:** 10.3390/molecules29153645

**Published:** 2024-08-01

**Authors:** Marta Radaelli, Elisa Scalabrin, Marco Roman, Gabriella Buffa, Irene Griffante, Gabriele Capodaglio

**Affiliations:** 1 Department of Environmental Sciences, Informatics and Statistics, University Ca’ Foscari Venice, Via Torino 155, 30172 Venezia, Italy; marta.radaelli@unive.it (M.R.); marco.roman@unive.it (M.R.); buffag@unive.it (G.B.); irene.griffante@gmail.com (I.G.); 2 CNR-ISP, Via Torino 155, 30172 Venezia, Italy

**Keywords:** elemental composition, ancient cereals, major and trace nutrients, intensive agriculture, organic agriculture

## Abstract

According to their nutritional value, their ability to adapt to the various environmental conditions, and their versatility, cereals are among the most cultivated plants in the world. However, the ongoing climate changes subject crops to important environmental stress that for some varieties leads to high production losses. Therefore, the selection of species and varieties that are more versatile and adaptable to different environmental conditions can be important. However, the characteristics of some cereals are not completely known; this is a priority before aiming to improve their cultivation. The aim of this study is to characterize select species that are potentially suitable for local environmental conditions and that possess nutritional value. The elemental composition was assessed in different cereal species grown following intensive and organic agriculture practices. Six species were grown for this study with techniques of intensive agriculture: *Triticum monococcum* L., *Triticum dicoccum* L., *Triticum aestivum* L., variety *Verna*, *Triticum durum* Desf., variety *Senatore Cappelli*, *Triticum durum* Desf., variety *Claudio*, and *Avena strigosa* Schreb.; four of these were also grown following organic procedures: *Triticum monococcum* L., *Triticum dicoccum* L., *Triticum aestivum* L., variety *Verna*, and *Triticum durum* Desf., variety *Senatore Cappelli*. The study considered twenty elements, including major nutrients (Ca, K, Mg, P, and S), seven micronutrients (B, Cu, Fe, Mn, Mo, Se, and Zn), and trace elements with toxic properties (Al, Ba, Cd, Cr, Na, Rb, Sc, and Sr) that can be accumulated at the seed level. The results highlight the differences in the element concentrations in the cereal seeds in relation to the genus and species; the highest concentrations of the major nutrients appeared in *T. monococcum*; the concentrations were 6.9, 2.09, 7.2, and 2.9 mg/g for K, Mg, P, and S, respectively. The highest concentrations of certain micronutrients, B, Ca, Mo, and Se (16, 785, 3.69, and 0.34 μg/g), were in *A. strigosa*. There is also evidence that the element content can be affected by the adopted cultivation procedure; however, the effects of the growing procedure can be significantly different when different species are considered. *T. monococcum,* grown by an organic procedure, presented lower concentrations of the major nutrients, while it demonstrated a modest increase in the micronutrients in the *T. durum* variety organic *S. Cappelli*, and the production procedure did not affect the elemental composition of the *T. aestivum* variety *Verna*. The survey also highlights that the studied species and the growing procedure affected the capacity to accumulate and translocate trace hazardous elements for human health at the seed level.

## 1. Introduction

Plants comprise most of the food consumed all over the world, and cereals represent 43% of the world’s food supply [1]. About half of all the cultivated areas in the world are used for cereal production, which forms the basis of human nutrition on all the continents, contributing to the energy and nutrient requirements of the world’s population.

The estimated world cereal production in 2023 was 2839 million tons, with an increase of 1.1% compared to 2022 [2].

Maize (*Zea mays*, L.) is the world’s leading produced cereal, with a harvest of more than 1163 million tons in 2022, followed by wheat (*Triticum* L., 808 million tons), and rice (*Oryza* L., 776 million tons); the sum of these three cereals represents about 90% of the world’s cereal production [3].

The need to feed as many people as possible and the pursuit of productivity and performance by intensive agriculture have resulted in the loss of many nutritional and taste properties in crops by privileging only a few cereal species and varieties [4]. This biodiversity loss has also greatly impacted the agricultural practices, with the increased need to use water, pesticides, and fertilizers [5]. Indeed, each species, has different optimal environmental conditions regarding growth and presents different responses to environmental stresses, both biotic and abiotic [6]. Increasing evidence indicates that agricultural production, as an important field related to food security, is becoming extremely vulnerable to climate change, and the need to favor sustainable cereals has been rising [7].

The ongoing climate changes, which modify the rainfall and temperature, subject crops to important environmental stress that for some varieties leads to high production losses [8]. This phenomenon is more evident for some hybrid varieties normally used in intensive production that have high productivity at the expense of stress tolerance [9]. Therefore, because climate change substantially influences the ecological environment, it has become a great challenge to select varieties that are able to adapt to varying environmental conditions. 

Ancient varieties, due to their adaptation that took place over a long time, proved to be more versatile than the main cereals and demonstrated high tolerance to various biotic and abiotic stresses [6,9]. In fact, because they are more resistant to temperature variations and require less water and fertilizer, they would reduce the impact of agriculture on the environment [10,11,12]. The removal of specific genes in crop species to improve their genetic pattern is the main cause of the enhanced resistance of some ancient grain species against biotic stresses [13].

Moreover, ancient grains could be the answer to the current state of food security, meeting the nutritional needs of a growing population and satisfying individuals with special nutritional needs, such as those with a sensitivity to proteins such as gluten [14,15]. 

Our dependence on only a few crop species limits our ability to meet the challenges posed by the negative effects of climate change and the consequences of food imbalance. As indicated in several studies, ancient cereal varieties might have beneficial nutritional profiles, and consumers appreciate the taste and flavor of the foods based on these products [16]. Moreover, ancient cereals have the potential to contribute to the improved sustainability and resilience of cropping systems by adapting to a wide range of soil conditions and requiring fewer cultivation inputs [7]. In addition, the production and consumption of foods based on ancient cereals indirectly encourage biodiversity, which has become a priority in environmental and organic farming circles. There is evidence that some traditional food crops, such as Tef [(*Eragrostis tef* (Zucc. Trotter)], are particularly nutritious and are also more resilient to marginal soil and climate conditions than the major cereals [10]. Therefore, due to their unique nutritional value and phytochemical profiles, as well as their sensory characteristics, there is good potential for ancient cereals and their associated products to become part of a healthy diet [17]. Studies carried out on *Triticum turgidum* ssp. *dicoccoides* (wild emmer) showed high concentrations of micronutrients, Fe, and Zn, significantly exceeding those of cultivated wheat [18]. High levels of Ca, Cu, and K were also measured in ancient *Triticum* species [19].

However, given the low or irregular performance of ancient cereal production, careful research in this field becomes a priority to improve ancient cereals’ cultivation. This has necessitated an alternative and robust approach to improve their resilience to diverse types of stresses and to increase the crop yields.

The aim of this study is to characterize selected cereal species that are potentially suitable for various environmental conditions, in terms of resistance, productivity, and nutritional value, to reduce the use of pesticides and human inputs and to identify a nutritionally superior food source. 

Different varieties of cereals, ancient grains and otherwise, were characterized from the chemical–nutritional point of view, determining the content of the nutrient elements. These parameters are of particular interest to investigate plants used both as food and forage [20,21,22,23].

The differences in the elemental composition were initially assessed in commercial samples to highlight the main differences because of the element translocation capability of plants, and the overall concentration in each tissue is generally species-specific [24]. Then, since the element composition may be due to differences in the soil composition and agronomic production techniques, the study was extended to a larger number of species grown in the open field in a pedologic homogeneous area. In addition, the species were subjected to different agricultural practices (conventional and organic) to highlight the differences related to the different production procedures.

The survey also evaluated the content of elements to verify the differences in the studied species to accumulate and translocate the trace elements of particular interest because phytotoxic elements are hazardous to human health [25,26,27].

## 2. Materials and Methods

### 2.1. Sample Cultivation

Thirteen samples were analyzed for element content. Three commercial samples were analyzed: einkorn wheat (*Triticum monococcum* L.), oats (*Avena sativa* L.), and spring wheat (*Triticum aestivum* L.). Six species were grown for this study with techniques of intensive agriculture: *Triticum monococcum* L. (FM), *Triticum dicoccum* L. (FD), *Triticum aestivum* L., variety *Verna* (V), *Triticum durum* Desf., variety *Senatore Cappelli* (SC), *Triticum durum* Desf., variety *Claudio* (C), and *Avena strigosa* Schreb. (A); four of the previous species were grown with organic agriculture: *Triticum monococcum* L. (FMB), *Triticum dicoccum* L. (FDB), *Triticum aestivum* L., variety Verna (VB), and *Triticum durum* Desf., variety Senatore Cappelli (SCB). The seeds were obtained from the Rural Seed Network of Umbria and from Los Prados Company. The seeds of the tested varieties were stored at 18 °C and 40% humidity in the storage cell of the University Ca’ Foscari. The growth studies following organic procedure were conducted in one greenhouse at the Department of Environmental Sciences, Informatics and Statistics, Ca’ Foscari University of Venice. Open field growth following conventional procedures was carried out at the experimental station of La Battistei Farm (winter wheat season 2018–2019), located in Cittadella Municipality, 45.674 N, 11.737 E, and 70 m elevation. The average annual rainfall from 1951 to 2014 at the station was 1429 mm, the average seasonal air temperature was 13.0 °C, and the average sunshine duration was 2978.72 h. The soil was ploughed to a depth of 20 cm with a tractor-drawn disc plough and large clods of soil were smoothed with a harrow to create a level seedbed. The sowing was carried out on 25 October 2018. The identified varieties were sown with double replication in plots of 5 m^2^ for both conventional and organic methods. Traditional (*Triticum monococcum* L., *Triticum dicoccum* L., *Triticum durum* Desf., variety *Senatore Cappelli*, *Triticum aestivum* L., variety *Verna*, and *Avena strigosa* Schreb.) and modern (*Triticum durum* Desf., variety *Claudio*) varieties were sown with 250 and 400 seeds per mm^2^, respectively. During the growing season, particularly on 18 February 2019, 30 g of ammonium nitrate (26% N) for *Triticum durum* Desf., variety *Claudio* and 15 g for the traditional varieties were applied to plants grown using the conventional method. On the same day, 60 g of Super Robur (15%) for Claudio and 30 g for the traditional varieties were applied to the organically grown varieties. Nitrogen fertilizer, however, was not administered to both einkorn and emmer as it is not necessary for cultivation but on the contrary increases the tendency to lodging. To prevent fungal infections, the varieties were sprayed with tetraconazole on 18 March 2019 for the intensively grown varieties and organic fungicide for the organic test. The conventional method tests used a dicotyledonous herbicide for seasonal weed control, while the organic tests used mechanical weed control. The harvest took place on 1 July 2019.

Samples were crushed manually in a mort and then milled more finely until a grain size of 5 mm by a ball mill (MM 400, Retsch, Verder Scientific, Haan, Germany) using Teflon gyres at a frequency of 28 Hz for 15 min (2 cycles). Every sample was obtained by milling at least 30 seeds.

### 2.2. Elements’ Determination

The commercial samples einkorn wheat, spring wheat, and oats (*T. monococcum*, *T. aestivum*, and *A. sativa*), which were used to optimize the analytical methods, were analyzed to determine major nutrient elements Ca, K, Mg, P, and S and trace elements Al, B, Ba, Cu, Fe, Li, Mn, Na, Rb, Se, Sr, and Zn. Samples of FM, FMB, FD, FDB, SC, SCB, C, V, VB, and A were analyzed to determine the same major nutrient elements. Li was excluded because always present at concentration similar to or lower than the detection limit. Mo was included because it is one micronutrient of particular interest; between the toxic trace elements, Cd and Cr were also determined for their environmental interest. 

Samples were digested following the procedure previously described in detail [28]. In short, digestion was carried out through a microwave oven (Ethos Milestone Srl, Torre Boldone (BR), Italy) as follows. One hundred mg of sample were placed in vessels of TFM (Modified Poly-Tetra-Fluoroethylene) with 6 mL of ultrapure HNO_3_ (ROMIL Ltd.) and 4 mL of ultrapure H_2_O_2_ (ROMIL Ltd., Cambridge, UK). The temperature control inside the vessels was carried out by means of a probe in one reference vessel.

Before each digestion cycle, vessels were subjected to a microwave washing cycle with 10 mL of HNO_3_ 65% suprapur (Merck, Darmstadt, Germany) according to the temperature and power program shown in Supplementary Materials, Table S1; at the end, vessels were rinsed by ultrapure water, reagent grade (ELGA, Lane End, High Wycombe, UK).

The element determination was carried out, after the procedure validation, by ICP-OES or ICP-MS depending on concentration levels.

#### Methodology Optimization for the Element Determination

The applied analytical methodologies were ICP-OES and ICP-MS, which were optimized by analyzing a certified reference material (apple leaves SRM-1515, NIST) and commercial cereal samples. 

The measurements by ICP-OES (iCap 7000 series, Thermo Scientific, Waltham, MA, USA) involved the optimization of the plasma conditions: the geometry of the plasma for signal acquisition in both axial and radial mode, and the selection of wavelength to minimize spectral interference; the optimal conditions are reported in the Supplementary Materials, Table S2. 

The analytical methodology referring to the ICP-MS determinations has already been reported Ranaldo et al., 2015 [28], and here it was adapted to a different instrumentation (iCap RQ ICP/MS, Thermo Fisher Scientific, Waltham, MA, USA), with QCell reaction cell; the instrumentation was calibrated by a tuning procedure conducted using a solution of Ba, Bi, Ce, Co, ln, Li, and U at a concentration of 1 g/L in HNO_3_ 2%. It considered the alignment of the torch, the velocity of the peristaltic pump, the cooling Ar flow and the auxiliary gas flow, the Ar flow for the nebulizer, the power transmitted to the torch, and the difference in potential applied to the ionic lenses; the optimal conditions are reported in the Supplementary Materials, Table S3. After the tuning procedure, preliminary performance tests were conducted to verify repeatability, both in standard acquisition mode (STD) and by applying the Qcell reaction cell (KEDS); based on these preliminary tests, to avoid any isobaric interference, it was decided to always use the KEDS procedure with He as reaction gas.

Quantification was carried out by external calibration curves using multi-element standard solutions obtained by dilution of single-element standard solution of 1000 mg/L. The determination coefficient (R^2^) obtained by ICP-OES for Al, B, Ba, Ca, Fe, K, Na, and P was always higher than 0.999, while, for Cu, Mg, Mn, Rb, and Zn, it was always higher than 0.99; the R^2^ values for calibration curves obtained by ICP-MS were always higher than 0.999 for all the elements.

The analytical performance of both the methodologies was assessed in terms of precision and accuracy (recovery %). Repeatability was assessed by repeating the measurements 4 times on the same solution obtained from the certified material SRM-1515. Reproducibility was estimated by the analysis of the commercial samples, and measurements were replicated 5 times on independent portion of each sample. 

Table 1 reports the concentrations, uncertainty, estimated as confidence interval from 4 measurements and a = 0.05, and recovery obtained by the two techniques on the certified reference material; accuracy (recovery %) is calculated as ratio % between the experimental results and the certified values. Table 2 and Table 3 report the concentrations obtained by analyses of the 3 commercial samples, using ICP-OES and ICP-MS, respectively; the standard deviation is calculated from 5 measurements carried out on independent portions of the same sample and represents the reproducibility of the method. 

On the basis of the performance of the two techniques and considering the expected concentration ranges for the various elements, the determinations were conducted by ICP-OES for the elements Al, Ca, Fe, K, Mg, Na, P, and S and by ICP-MS for B, Ba, Cu, Mn, Rb, Se, Sr, and Zn. Since the matrix is particularly complex, to avoid memory effects, the sample loading sequence involved a washing procedure by means of HNO_3_ 2% after each sample. 

### 2.3. Data Elaboration and Statistical Analysis

Statistical analyses were carried out by Metaboanalyst 6.0 [29] https://www.metaboanalyst.ca/ (accessed on 11 April 2024). After data normalization, ANOVA test was applied to verify the significance of the differences in the mean values of each genotype, considering the *p*-value significance at level < 0.01. Hierarchical cluster analysis was performed using Ward’s method and evaluating squared Euclidean distance. The number of clusters was determined in accordance with the Pseudo T-Squared grouping criterion.

A principal component analysis (PCA) was also applied to emphasize differences among plant groups based on the elemental composition.

## 3. Results and Discussion

### 3.1. Commercial Samples

The multi-element analysis on the commercial samples was conducted to assess the differences in the composition between different genera and species; two genera were considered, oats and wheat (*Avena sativa* and *Triticum,* respectively), and two species of the same genus *Triticum* (*T. monococcum* and *T. aestivum*) were used as models.

**Figure 1:**
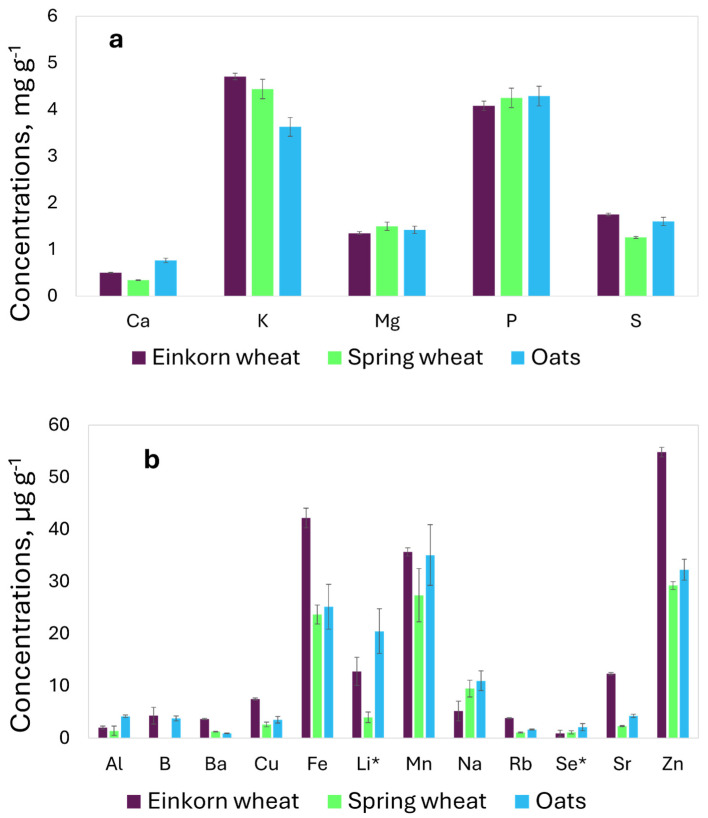
Element concentrations in commercial samples of einkorn wheat (*T. monococcum*), spring wheat (*T. aestivum*), and oats (*A. sativa*): (**a**) major elements in mg g^−1^; (**b**) trace elements in μg g^−1^, Li*, and Se* μg × 100 g^−1^.

The elemental composition is differentiated in the major elements and trace elements in relation to their concentrations, and the results are reported in Figure 1a, for the major elements, and in Figure 1b, for the trace elements.

Tests were carried out to highlight the significant differences between the genera (*Avena* and *Triticum*) and between the two species of the genus *Triticum* (*T. monococcum* and *T. aestivum*); the element concentrations were considered significantly different if the *p*-value was lower than 0.01. The results did not show differences between the genera and species for the content of P and Mg, while the mean concentration of K was significantly lower in the oats (*A. sativa*), and the *t*-test did not show a significant difference between the two species of genus *Triticum*. The concentration of sulfur (S) in the oats and einkorn wheat *(T. monococcum*) was comparable, while it was significantly lower in the spring wheat (*T. aestivum*). The content of Ca was significantly different in relation to the genus and species; the highest concentration was observed in the oats, and the differences were also significant between the two species of *Triticum*, with lower concentrations in the spring wheat. 

The major difference observed in the commercial samples is the higher levels of Ba, Cu, Fe, Rb, Sr, and Zn in the einkorn wheat samples in comparison to the spring wheat and oats, as previously highlighted by some studies [19,30,31]. The concentration of Na (5.2 μg g^−1^) in the einkorn wheat was about half the content in the oats and wheat (11 and 9.5 μg g^−1^, respectively).

### 3.2. Samples Grown in Open Field by Conventional and Organic Techniques

The differences in the elemental composition of the samples may be due to differences in the soil composition and agronomic techniques; therefore, the study was extended to samples grown in an open field in a homogeneous area from the pedologic point of view. The study was applied to a higher number of *Triticum* species (*T. monococcum*, *T. dicoccum*, *T. durum-Senatore Cappelli*, *T. durum-Claudio*, and *T. aestivum-Verna*) and, to highlight the differences related to the production techniques, the *T. monococcum*, *T. dicoccum*, *T. durum*-*Senatore Cappelli*, and *T. aestivum-Verna* species were also produced by organic agriculture.

**Figure 2:**
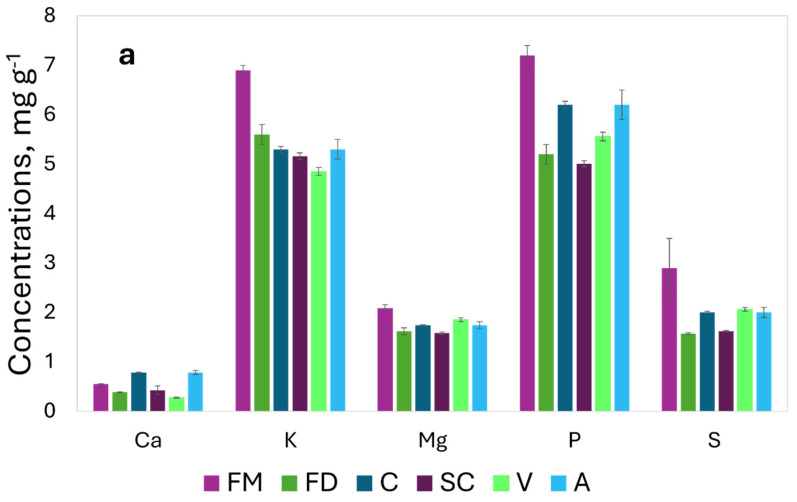

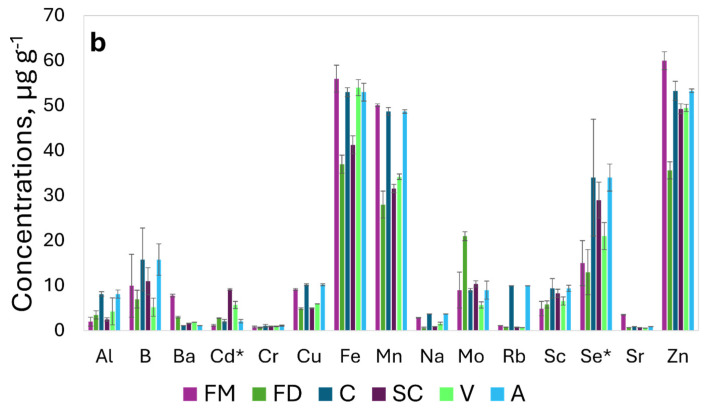
Element concentrations in samples of various *Triticum* species and *Avena strigosa*: (**a**) major elements in mg g^−1^; (**b**) trace elements in μg g^−1^, Cd*, and Se* μg × 100 g^−1^.

The survey evaluated the content of the additional elements (Cd, Cr, Mo, and Sc) to verify the differences among the studied species for their ability to accumulate trace elements of particular interest at the seed level; Mo is an important trace element because it is a cofactor in enzymatic systems [22,23]; Cd, Cr, and Sc are phytotoxic elements and are hazardous to human and animal health [25,26]. The element composition of the cereals grown by conventional agriculture is reported in Figure 2 (Figure 2a for the major nutrients and Figure 2b for the trace elements).

The mean concentrations and the standard deviations obtained by three measurements on the independent samples are reported in Table 4.

The element concentration detected in the samples grown in an open field are comparable to the recent literature data, as reported in Table 5; in general, in this study, we observed higher concentrations of B, K, and P in comparison to the previously reported values. The literature data of the Se concentration are variable, and our results are consistent with the highest concentrations, while lower concentrations were observed for Na with respect to the literature data [18,32,33,34,35,36,37,38,39]. 

The highest concentrations of the major nutrients were observed in the FM samples, while the highest concentrations of many minor nutrients (Cu, Fe, Mn, Mo, and Zn) were detected both in FM and A.

### 3.3. Evaluation of Element Content by Statistical Analysis

**Figure 3:**
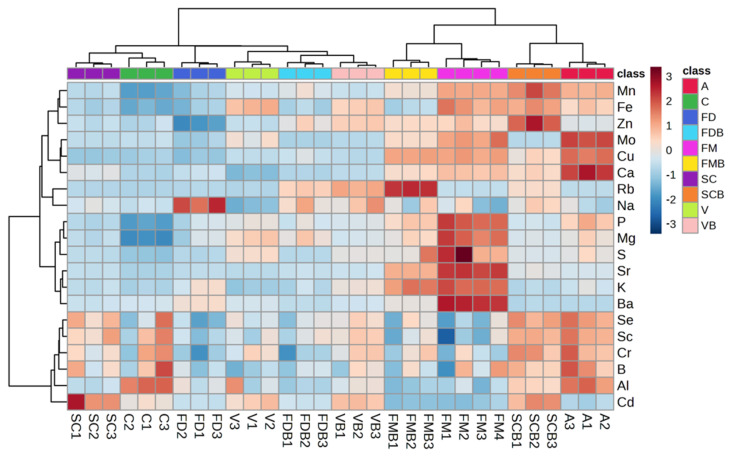
Heatmap of elements in crop seed samples. The color-coded matrix represents the intensities of the element concentration in each sample. Each sample is marked by the class code and the sample number; for the class codes that identify the species, see the text.

To highlight the differences between the genera, species, and in relation to the growing techniques, a cluster analysis was applied, both on the samples and elements, assessed by a heatmap plot (Figure 3). The results highlight that FM appears to be particularly enriched in nutrients, with the highest levels of the major nutrients and average concentrations of trace nutrients higher than in the other *Triticum* species. In the FM samples, only the Se and B concentrations demonstrated to be lower than the values determined in A and the other *Triticum* species. The concentrations of the major and trace nutrients were regularly lower in *T. durum*, of both varieties *Claudio* and *Senatore Cappelli* (C and SC), when grown using the conventional procedures; only B and Se and other non-essential elements (Sr, Cr, Cd, and Al) were enriched in this species. The *Triticum* species presenting the lowest concentrations of nutrients are *T. dicoccum* (FD) and *T. aestivum Verna* (V) when grown by the conventional procedures. Sample A (*A. strigosa*) proved to be characterized by particularly high levels of Mo, Cu, and Ca, while SCB demonstrated the highest levels of Mn, Fe, and Zn.

The growing procedure affects the element content in *T. monococcum* and *T. durum Senatore Cappelli*; indeed, FMB presents a lower content of nutrients, contrary to the results obtained for SCB, which showed higher concentrations of some trace nutrients (Mn, Fe, Cu, and Se) in comparison to the SC samples. Previous studies showed different trends in the comparison of nutrients between organic and conventional cultivated crops depending on the species. Ciolek et al., 2012 [40] showed increases in Ca and Mg in conventional oats while reporting decreases in Ca, K, Mg, Fe, and Zn in conventional wheat. 

Limited differences in the nutrient concentrations were observed between V and FD when the conventional or organic growing procedures were applied.

**Figure 4:**
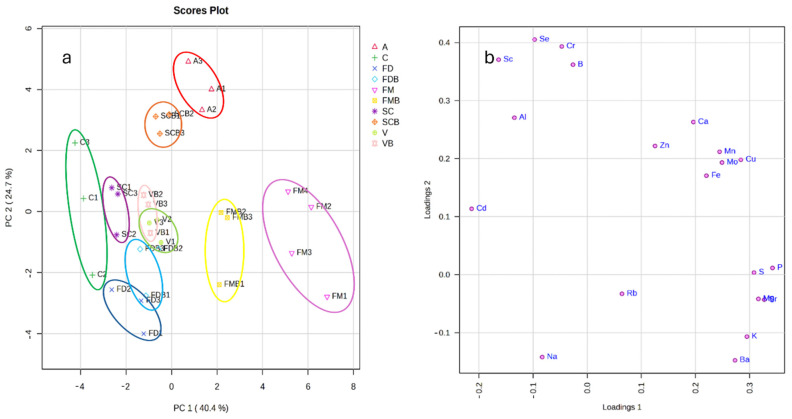
Principal component analysis of data for the element characterization of cereals. (**a**) Score plot of the 30 samples; (**b**) loading plot based on the same data set.

PCA was applied to highlight the grouping of the samples; the explained variance by the first and second components was 40% and 25%, respectively (Figure 4a,b). The score plot principally revealed the separation of the FM and FMB samples by the first component and a partial separation of A, C, and SC, while the second component highlighted the separation of the A samples and SCB. FD and FDB were only partially separated by the second component. No significant differences were observed between V and VB, while limited differences were highlighted between FD and FDB.

The first component, high loadings, responsible for the separation of FM and FMB from the other species, is mainly related to the major nutrients (S, Mg, P, and K), which showed high concentrations in these samples (Figure 2a). The C samples were characterized by the variable Cd, which showed the lowest loading value in the first component.

The second component explains the contribution of B, Se, and some elements that are not essential for biological systems (Al, Cr, and Sc), with the highest values in the A and SCB samples; the other trace nutrients (Mn, Fe, Cu, Mo, Ca, and Zn) contribute to both the components.

Therefore, it can be stated that the differences in the element concentrations in cereal seeds are related to the genus and species; however, there is evidence that the element content can also be affected by the adopted cultivation process; indeed, the results show that different cultivation procedures can affect the element composition. However, it must be highlighted that the effect of the growing procedure can be significantly different when different species are considered, resulting in opposite trends; the results show, indeed, that the production of FMB decreases the concentrations of the major nutrients, while SCB is richer in trace nutrients with respect to the same species grown by the conventional procedures. The effect of fertilization on the concentrations of trace nutrients was studied. Stroud et al. [41] studied the effect of S fertilization on the Se and Mo concentrations, emphasizing the decreases in Se and Mo when S fertilization was applied. The growing procedures regarding other species compositions (*T. aestivum* variety *Verna* or *T. dicoccum*) seem not to have effects or have negligible effects.

## 4. Conclusions

The elemental composition was assessed in different cereal species grown following conventional and organic procedures. Six species were grown for this study with techniques of conventional agriculture: *Triticum monococcum* L., *Triticum dicoccum* L., *Triticum aestivum* L., variety *Verna*, *Triticum durum* Desf., variety *Senatore Cappelli*, *Triticum durum* Desf., variety *Claudio*, and *Avena strigosa* Schreb.; four of these were also grown with organic agriculture: *Triticum monococcum* L., *Triticum dicoccum* L., *Triticum aestivum* L., variety *Verna*, and *Triticum durum* Desf., variety *Senatore Cappelli*.

The study considered twenty elements, including major nutrients (Ca, K, Mg, P, and S), seven micronutrients (B, Cu, Fe, Mn, Mo, Se, and Zn), and trace elements with toxic properties (Al, Ba, Cd, Cr, Na, Rb, Sc, and Sr) that can be accumulated at the seed level.

The results highlight the differences in the element concentrations in the cereal seeds in relation to the genus and species. The highest concentrations of the major nutrients were found in FM, while the highest concentrations of some micronutrients, such as B and Se, and some toxic elements (Cr, Sc, and Al) were in A. FM therefore seems to be interesting from the nutritional point of view, showing high content in terms of the major elements and high levels of other important nutrients, such as Mn, Fe, and Zn. The presence of potentially toxic elements in the seeds, as observed in A and SCB, should be carefully considered and needs to be evaluated via further study. There is also evidence that the element content can be affected by the adopted cultivation procedure; however, it must be highlighted that the effect of the growing procedure can be significantly different when different species are considered. FMB presented lower concentrations of the major nutrients, while the production procedure did not affect the elemental composition of the *T. aestivum* variety Verna; sometimes, the effect can also contrast among the same species, as observed for SCB, which resulted in some trace nutrients being richer in comparison to the conventional procedures.

The survey also highlights that the studied species and the growing procedure affect the ability to accumulate and translocate trace hazardous elements for human health at the seed level.

It must be borne in mind that the response of plants, such as the ability to assimilate the elements from the soil, is greatly influenced by the environmental conditions, as well as the climatic characteristics and soil composition; therefore, also taking into account the variability in the biological systems, the study is expected to be extended to different environmental plant growth conditions and to a higher number of samples.

## Figures and Tables

**Table 1:** Concentrations in μg/g of elements in reference material SRM 1515, certificated value ± tolerance interval (TI); experimental results by ICP-OES and ICP-MS with mean value ± confidence interval and recovery as ratio between the result and the certified value % (R%).

Element	Cert Conc ± TI	ICP-OES Conc ± CI	R%	ICP-MS Conc ± CI	R%
Al	284.5 ± 5.8	216.3 ± 4.7	76	182 ± 13	64
B	27.6 ± 2.8	---		27.9 ± 1.9	101
Ba	48.8 ± 2.3	62.7 ± 0.8	128	47.4 ± 0.6	97
Ca *	15.250 ± 0.100	16.08 ± 0.05	105	---	
Cu	5.69 ± 0.13	6.3 ± 0.2	112	6.0 ± 0.1	105
Fe	82.7 ± 2.6	74.7 ± 0.7	90	81.5 ± 8.2	99
K *	16.080 ± 0.210	17.1 ± 0.2	106	11.7 ± 0.8	73
Mg *	2.710 ± 0.120	3.00 ± 0.04	111	2.71 ± 0.12	100
Mn	54.1 ± 1.1	58.7 ± 0.2	108	56.2 ± 2.4	104
Na	24.4 ± 2.1	22.5 ± 2.0	92	---	
P *	1.593 ± 0.068	1.65 ± 0.19	107	---	
Rb	10.2 ± 1.6	---		9.8 ± 0.2	96
Sr	25.1 ± 1.1	28.9 ± 0.2	115	26.2 ± 0.5	105
Zn	12.45 ± 0.43	12.2 ± 0.1	98	10.5 ± 0.4	84

* Concentration in mg/g.

**Table 2:** Concentrations in µg/g and reproducibility as standard deviations by 5 measurements on independent samples (SD) of elements in commercial samples of spelt (*Triticum monococcum*), wheat (*Triticum aestivum*), and oats (*Avena sativa*), determined by ICP-OES.

	Spelt		Wheat		Oats	
Element	Mean	SD	Mean	SD	Mean	SD
Al	--	--	--	--	--	--
B	--	--	--	--	--	--
Ba	4.88	0.08	2.19	0.14	2.67	0.20
Ca *	0.505	0.008	0.345	0.009	0.764	0.049
Cu	8.83	0.13	4.11	0.19	5.01	0.46
Fe	41.2	0.8	38.8	1.9	38.0	2.6
K *	4.71	0.07	4.44	0.21	3.63	0.20
Li	--	--	--	--	--	--
Mg *	1.35	0.03	1.50	0.09	1.42	0.08
Mn	39.6	0.8	43.6	1.6	51.6	2.8
Na	5.2	1.9	9.5	1.6	11.0	1.9
P *	4.08	0.10	4.25	0.21	4.29	0.21
Rb	--	--	--	--	--	--
S *	1.75	0.03	1.26	0.02	1.60	0.09
Se	--	--	--	--	--	--
Sr	12.35	0.23	2.30	0.10	4.28	0.29
Zn	54.84	0.91	29.26	0.75	32.3	2.0

* Concentration in mg/g.

**Table 3:** Concentrations in µg/g and reproducibility as standard deviations by 5 measurements on independent samples (SD) of elements in commercial samples of spelt (*Triticum monococcum*), wheat (*Triticum aestivum*), and oats (*Avena sativa*), determined by ICP-MS.

	Spelt		Wheat		Oats	
Element	Mean	SD	Mean	SD	Mean	SD
Al	2.01	0.33	1.4	0.9	4.19	0.24
B	4.3	1.6	udl		3.78	0.47
Ba	3.66	0.12	1.26	0.08	0.93	0.07
Ca	--	--	--	--	--	--
Cu	7.49	0.20	2.62	0.44	3.52	0.64
Fe	42.2	1.9	23.7	1.8	25.2	4.3
K *	4.90	0.06	3.33	0.64	2.97	0.45
Li	0.13	0.03	0.04	0.01	0.21	0.04
Mg *	1.25	0.03	0.94	0.17	1.02	0.15
Mn	35.7	0.8	27.4	5.1	35.1	5.8
Na	--	--	--	--	--	--
P	--	--	--	--	--	--
Rb	3.84	0.09	1.08	0.10	1.65	0.09
S	--	--	--	--	--	--
Se **	9	6	11	3	21	7
Sr	10.89	0.26	1.59	0.15	2.79	0.40
Zn	48.79	0.89	17.9	3.2	25.2	1.8

* Concentration in mg/g; ** concentration in ng/g; udl = under detection limit.

**Table 4:** Element concentrations in μg/g, mean values, and standard deviations (in brackets) in the samples grown following extensive and organic agriculture.

	Triticum									Avena
Element	Monococ.	Monococ. Organic	Dicoccum	Dicoccum Organic	Durum (*Claudio*)	Durum (*S. cappelli*)	Durum Org. (*S. cappelli*)	Aestivum (*Verna*)	Aestivum Org. (*Verna*)	Strigosa
Al	2 (1)	1.2 (0.6)	3.5 (0.9)	1.8 (0.4)	8.6 (0.5)	2.5 (0.4)	5.2 (0.3)	4 (3)	3.9 (0.6)	8.2 (0.9)
B	10 (7)	7.5 (3.5)	6.8 (2.3)	7.1 (2.2)	13 (6)	11 (3)	12.7 (1.4)	5.2 (2.0)	8.88 (3.5)	16 (3)
Ba	7.8 (0.3)	2.5 (0.02)	3.0 (0.2)	1.3 (0.1)	1.81 (0.03)	1.58 (0.08)	1.1 (0.1)	1.87 (0.03)	1.73 (0.05)	1.13 (0.02)
Ca	550 (9)	495 (9)	384 (9)	343 (3)	340 (5)	426 (9)	504 (17)	275 (9)	364 (3)	785 (40)
Cd *	12 (2)	9 (1)	28 (1)	30 (1)	45 (4)	92 (2)	75 (10)	57 (8)	62 (3)	21 (4)
Cr	0.8 (0.2)	0.9 (0.2)	0.7 (0.1)	0.7 (0.1)	1.0 (0.3)	0.93 (0.07)	1.13 (0.09)	0.93 (0.09)	0.98 (0.09)	1.1 (0.1)
Cu	9.2 (0.2)	9.1 (0.1)	4.9 (0.2)	6.3 (0.3)	5.3 (0.2)	5.0 (0.1)	7.55 (0.5)	5.93 (0.05)	5.74 (0.08)	10.2 (0.2)
Fe	55 (3)	41.7 (1.5)	37 (2)	39.7 (1.5)	35.2 (1.0)	41 (2)	59.3 (2.4)	54.0 (1.8)	50.8 (1.3)	53.1 (0.9)
K **	6.9 (0.1)	6.6 (0.2)	5.6 (0.2)	5.1 (0.2)	5.00 (0.06)	5.16 (0.07)	5.09 (0.050)	4.85 (0.08)	5.63 (0.04)	5.34 (0.15)
Mg **	2.09 (0.07)	1.76 (0.06)	1.62 (0.07)	1.76 (0.08)	1.30 (0.02)	1.58 (0.02)	1.66 (0.01)	1.85 (0.04)	1.65 (0.01)	1.74 (0.07)
Mn	50.1 (0.3)	40.1 (0.5)	28 (3)	37.4 (2.7)	20.3 (0.9)	31.6 (0.9)	57.4 (4.1)	34.2 (0.6)	33.1 (0.5)	48.7 (0.4)
Mo	2.84 (0.09)	1.83 (0.03)	0.6 (0.2)	0.69 (0.02)	1.00 (0.06)	0.93 (0.01)	0.86 (0.03)	1.6 (0.3)	0.855 (0.008)	3.69 (0.07)
Na	9 (4)	11.0 (3.7)	21 (1)	13.7 (2.6)	9.4 (0.4)	10.4 (0.7)	12.9 (1.6)	5.7(0.7)	14.6 (2.6)	9 (2)
P **	7.2 (0.2)	6.0 (0.2)	5.2 (0.2)	5.4 (0.2)	4.20 (0.07)	5.01 (0.06)	5.28 (0.04)	5.56 (0.09)	5.39 (0.05)	6.2 (0.3)
Rb	1.12 (0.06)	7.4 (0.1	0.78 (0.05)	3.4 (0.2	0.63 (0.02)	0.74 (0.06)	2.84 (0.07	0.69 (0.04)	4.4 (0.2)	1.00 (0.03)
S **	2.9 (0.6)	2.2 (0.5)	1.57 (0.02)	1.58 (0.06)	1.36 (0.02)	1.62 (0.01)	1.81 (0.02)	2.06 (0.04)	1.90 (0.01)	2.0 (0.1)
Sc	4.9 (1.6)	6.3 (1.6)	5.8 (0.8)	6.8 (0.9)	8.1 (2.2)	8.3 (0.9)	9.0 (0.2)	6.6 (0.9)	8.0 (0.6)	9.4 (0.7)
Se	0.15 (0.05)	0.2 (0.1)	0.13 (0.05)	0.18 (0.05)	0.24 (0.13)	0.29 (0.04)	0.33 (0.02)	0.21 (0.03)	0.25 (0.03)	0.34 (0.03)
Sr	3.5 (0.1)	2.19 (0.01)	0.68 (0.03)	0.77 (0.04)	0.43 (0.02)	0.71 (0.02)	1.12 (0.07)	0.603 (0.004)	0.82 (0.04)	0.95 (0.01)
Zn	60 (2)	58.8 (0.3)	36 (2)	56.3 (3.8)	48.3 (2.1)	49 (1)	77.8 (5.2)	49.5 (0.8)	61.2 (0.6)	53.3 (0.4)

* Concentration in ng/g; ** concentration in mg/g.

**Table 5:** Literature data of element concentrations in cereals in μg/g.

Genus	Triticum													Avena	
Species	Wheat ^a^	Durum ^b^	Wheat ^c^	Aestivum ^d^	Wheat ^e^	Turgidum			Monocc ^g^	Aestivum	Aestivum ^h^	Durum ^h^	Tritordeum ^h^	Sativa ^a^	Sativa ^i^
Variety						Durum ^f^	Durum ^g^	Dicocc ^g^		Spelta ^g^					
Al															
B			2.85												
Ba															
Ca	380	350	477		415						454	446	434	520	540
Cd *				420											
Cr				2.46											
Cu		4.0		5.63	5.10										6.00
Fe	39	50.4	19.6	0.0	37.0	46	33.0	34.1	45.9	41.8				38.0	47.0
K **	3.40	4.78	3.33		4.20									3.50	4.29
Mg **	1.20	1.03	0.87		1.35	1.34					1.19	1.12	1.31	1.10	1.77
Mn			19.7	81.6	35.0										49.0
Mo															
Na	30.0		20.0			35.0								90.0	20.0
P **		3.93	3.06			3.65									5.23
Rb															
S **			0.84			1.08									
Sc															
Se *	20			160			80.8	229.3	278.9	209.0				30	
Sr															
Zn	29	36.8	28.8	25.7	38.0	61	21.2	22.8	22.4	22.9	31.6	34.3	36.5	33.0	40.0

* ng/g; ** mg/g; ^a^ [32]. ^b^ [33]. ^c^ [34]. ^d^ [37]. ^e^ [35]. ^f^ [18]. ^g^ [36]. ^h^ [38]. ^i^ [39].

## Data Availability

The original contributions presented in the study are included in the article (and Supplementary Materials), further inquiries can be directed to the corresponding authors.

## Supplementary Materials

The following supporting information can be downloaded at https://www.mdpi.com/article/10.3390/molecules29153645/s1, Table S1. Microwave oven program for seed digestion. Table S2. Instrumental parameters for the ICP-OES measurements. Table S3. Instrumental parameters for the ICP-MS measurements.

## Abbreviations

Conventional *Triticum monococcum* L. (FM), conventional *Triticum dicoccum* L. (FD), conventional *Triticum aestivum* L., variety *Verna* (V), conventional *Triticum durum* Desf., variety *Senatore Cappelli* (SC), conventional *Triticum durum* Desf., variety *Claudio* (C), conventional *Avena strigosa* Schreb. (A), organic *Triticum monococcum* L. (FMB), organic *Triticum dicoccum* L. (FDB), organic *Triticum aestivum* L., variety *Verna* (VB), and organic *Triticum durum* Desf., variety *Senatore Cappelli* (SCB).
