# Supplementary material for: Characterization of Ancient Cereals Cultivated by Intensive and Organic Procedures for Element Content
Source: Molecules. 2024 Aug 1;29(15):3645. doi: 10.3390/molecules29153645 (PMC11313877; doi:10.3390/molecules29153645)
Supplement: Supplementary file 1 [file molecules-29-03645-s001.zip › molecules-3069579-supplementary.pdf]

Article

Characterization of Ancient Cereals cultivated by intensive and organic procedures for elements content.

Marta Radaelli[1], Elisa Scalabrin[2*], Marco Roman[1], Gabriella Buffa[1], Irene Griffante[1**], Gabriele Capodaglio[1*]

[1]DAIS, University Ca' Foscari Venice, Via Torino 155, 30172 Venezia-Mestre, Italy
[2]CNR-ISP, Via Torino 155, 30172 Venezia-Mestre, Italy

*Correspondence: Capodaglio G., Scalabrin E., e-mail: capoda@unive.it; elisa.scalabrin@cnr.it;

**Present address: Cerba HealthCare Italia, via acquapendente 37/a, 35126 Padova, Italy.

Supplementary information

Table S1. Microwave hoven program for seeds digestion.

Time (min)	Power (W)	Temerature (°C)
15	1500	100
10	1500	120
10	1500	140
10	1500	160
15	1500	180

Table S2. Instrumental parameters for the ICP-OES measurements.

Parameter	value
Plasma RF Power	1150 W
Auxiliary argon	0.5 L/min
Cooling flow	12 L/min
Nebulizer argon flux	0.55 L/min
Nebulizer gas pressure	210 kPa
Peristaltic pump rate	50 rpm

Element	Torce geometry	Wavelength, nm
Al	Rad	396
B	Ax	208
Ba	Rad	455
Ca	Rad	422
Cd	Ax	285
Cu	Ax	324
Fe	Rad	259
Li	Ax	670
K	Rad	769
Mg	Rad	279
Mn	Ax	259
Mo	Ax	202
Na	Rad	589
P	Rad	213
Rb	Ax	780
S	Rad	180
Se	Ax	196
Sn	Ax	189
Sr	Rad	407
Zn	Ax	371

Table S3. Instrumental parameters for the ICP-MS measurements.

Parameter	Value
Plasma RF power	1550 W
Interface temperature	27° C
Cooling argon flow	0.68 L/min
Auxiliary argon flux	14 L/min
Cooling argon flux	0.8 L/min
Nebulizer argon flux	1.0 L/min
Peristaltic pump rate	40 rpm
Pirani pressure	1.7 mbar
Penning pressure	8 x 10^{-7} mbar
